# Comparative Effects of THC and CBD on Chemotherapy-Induced Peripheral Neuropathy: Insights from a Large Real-World Self-Reported Dataset

**DOI:** 10.3390/biomedicines13081921

**Published:** 2025-08-06

**Authors:** Ravit Geva, Tali Hana Bar-Lev, Lee Ahuva Lavi Kutchuk, Tali Schaffer, Dan Mirelman, Sharon Pelles-Avraham, Ido Wolf, Lihi Bar-Lev Schleider

**Affiliations:** 1 Division of Oncology, Tel Aviv Sourasky Medical Center, Tel Aviv 6423906, Israel; talibl@tlvmc.gov.il (T.H.B.-L.); leelavi@tlvmc.gov.il (L.A.L.K.); talisch@tlvmc.gov.il (T.S.); danmi@tlvmc.gov.il (D.M.); sharonpa@tlvmc.gov.il (S.P.-A.); idow@tlvmc.gov.il (I.W.); 2 Research Department, Tikun Olam–Cannabis Pharmaceuticals, Tel Aviv 6296602, Israel; lihibarlev@gmail.com

**Keywords:** cannabidiol (CBD), cannabinoid, cannabis, chemotherapy, neuropathy, side effects, quality of life

## Abstract

**Background/Objective:** Chemotherapy-induced peripheral neuropathy (CIPN) is a common dose-limiting adverse effect of various chemotherapeutic agents. Previous work demonstrated that cannabis alleviates symptoms of oxaliplatin-induced CIPN. To evaluate the effects of cannabis components, cannabidiol (CBD) and tetrahydrocannabinol (THC), on CIPN-related symptoms. **Methods:** We reviewed a patient-reported outcomes dataset from “Tikun Olam,” a major medical cannabis provider. Of 1493 patients, 802 reported at least one CIPN symptom at baseline, including a burning sensation, cold sensation, paresthesia (prickling) and numbness, and 751 of them met the study inclusion criteria. Patients were categorized into THC-high/CBD-low and CBD-high/THC-low groups. Symptom changes after six months of cannabis use were analyzed using K-means clustering and logistic regression, incorporating interactions between baseline symptoms and THC and CBD doses. Linear regression assessed changes in activities of daily living (ADL) and quality of life (QOL). **Results:** Both groups reported symptom improvement. The THC-high group showed significantly greater improvement in burning sensation and cold sensation (p = 0.024 and p = 0.008). Improvements in ADL and QOL were also significantly higher in the THC group (p = 0.029 and p = 0.006). A significant interaction between THC and CBD was observed for symptom improvement (p < 0.0001). **Conclusions:** Cannabis effectively reduces CIPN symptoms and improves QOL and ADL. Higher THC doses were more effective than lower doses, with combined CBD and THC doses yielding greater symptom relief.

## 1. Introduction

Chemotherapy-induced peripheral neuropathy (CIPN) is a common side effect of cumulative dosage [1], with considerable variability in symptoms and severity [2]. The estimated prevalence of CIPN varies between 30 and 40% [2] and reaches as high as 90% [1]. CIPN-related symptoms are felt primarily in the hands and feet, with numbness, paresthesia, shooting or stabbing pain, burning, cramping, and hypersensitivity to cold [3]. The mechanism of this condition involves neuronal toxicity and inflammation [2,4,5], with sensory symptoms tending to be more severe than motor or autonomic ones [2]. CIPN progresses in a dose-dependent manner [2,4] and may require dose reduction or discontinuation of the chemotherapy [2]. Management of these symptoms remains challenging due to the limited efficacy of existing interventions, such as prevention of therapy, reduction of dose, switching of therapies, time from end of treatment, and pharmacological therapies, which do not provide substantial benefit to most patients and are associated with significant side effects [4].

Opioids are currently considered the gold standard for the relief of cancer-related pain [6,7]. However, they are associated with adverse effects, such as constipation, sedation, addiction, and, in extreme cases, overdose [6]. The medicinal use of cannabis has a long history, but it became progressively more restricted in the 20th century. Recent legislation, however, has altered the legality of its use, with clinical trials now focusing upon its potential role in alleviating the side effects of cancer therapy [8].

**Figure 1:**
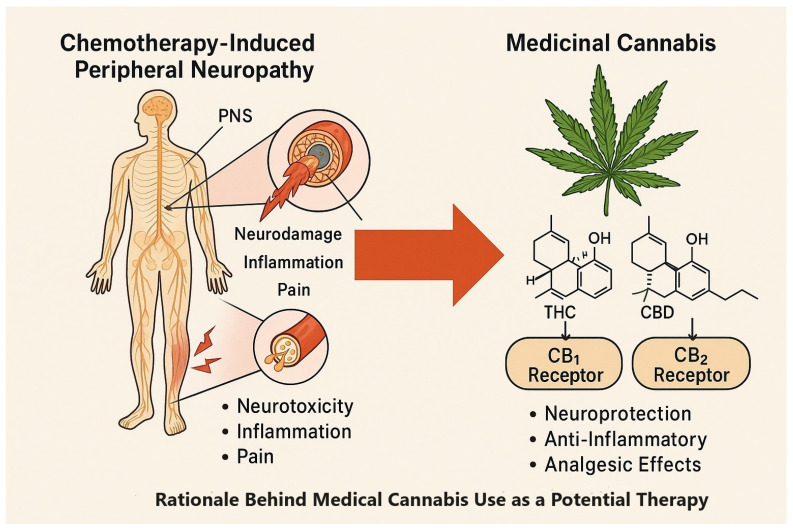
CIPN pathology and potential therapeutic targets of THC and CBD via CB1/CB2 receptors.

The *Cannabis sativa* plant contains many chemical compounds, including over 100 cannabinoids [7]. Cannabinoids have both desirable central and peripheral effects, providing analgesia, muscle relaxation, anti-inflammation, and anxiolysis without the risk of overdose. They can also enhance the effect of opioids and affect the expression of opioid receptors, leading to greater relief of pain and reduction in opioid use [6]. Tetrahydrocannabinol (THC) is the predominant psychoactive of the two primary cannabinoids [7,9], whereas cannabidiol (CBD) is a non-psychoactive cannabinoid believed to have multiple therapeutic benefits, although evidence-based corroboration is limited [7]. Cultivators have bred and selected specific cannabis strains based upon either a high THC content or high CBD content, resulting in vast diversity of legally available cannabis-based products [10]. These products are generally well tolerated, with mild and transient side effects, but the variation in efficacy between strains is not compatible with the medical model of drug prescription [11]. The precise mechanisms by which cannabinoids affect pain remain controversial, and clinical trials have shown inconsistent results [10,11,12,13] (Figure 1, Table 1), most likely due to variations in the studied substances [10]. The lack of standardization of cannabis treatment adds to the complexity since physicians must exercise discretion in determining appropriate use and patient suitability [14].

We had earlier investigated the effect of cannabis upon patients undergoing oncological treatment who sustained CIPN due to the administration of oxaliplatin [15]. The results of that investigation indicated a significant difference in CIPN levels between patients exposed to cannabis and those who were not (control group). The current study was conducted on a larger dataset and aimed to confirm our previous findings as well as to gain greater insight into the differential association between THC and CBD and CIPN symptoms. Specifically, we aimed to determine which cannabis component, THC or CBD, contributes to the alleviation of CIPN-related symptoms and whether there is a dose-dependent effect.

## 2. Materials and Methods

### 2.1. Study Design and Setting

This study is an analysis of a self-reported outcome dataset collected by Tikun Olam, a large medical cannabis provider in Israel [16,17]. The analysis aimed to evaluate the effects of medical cannabis, specifically THC and CBD, on symptoms related to chemotherapy-induced peripheral neuropathy (CIPN). The dataset included patients who had started medical cannabis treatment between 2009 and 2018 and completed patient-reported outcome (PROM), Activities of Daily Living (ADL), and Quality of Life (QOL) questionnaires both before initiating cannabis treatment and after six months of continuous use.

**Figure 2:**
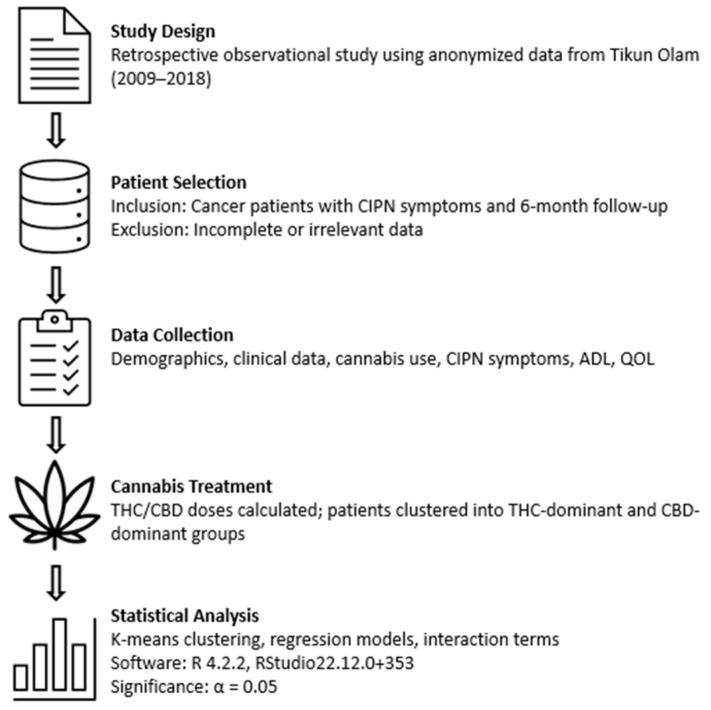
Study methodology flowchart outlining data selection, patient clustering, and statistical analysis.

A flowchart summarizing the study methodology, including data source identification, inclusion and exclusion criteria, patient clustering, and statistical analyses, is presented in Figure 2.

### 2.2. Participant Criteria

Patients were included based on the following criteria: (1) diagnosis of cancer and documented chemotherapy-induced symptoms, (2) at least one CIPN-related symptom at baseline (burning sensation, cold sensation, paresthesia, or numbness), and (3) complete PROM, ADL, and QOL data at baseline and after six months of cannabis use. We excluded patients whose records were incomplete or who failed to meet the reporting requirements

Out of 5063 consecutive medical records reviewed, 802 patients reported at least one CIPN-related symptom, and 751 met all inclusion criteria and were included in the final analysis.

### 2.3. Data Compilation

The analysis utilized self-reported patient data, including age (range 18–90 years), sex, height, weight, and body mass index (BMI), as well as disease duration and comorbidities. Of the 802 patients included in the study, 448 were female, and 303 were male (51 patients had missing or unclassified sex data). Lifestyle factors (e.g., smoking status, physical activity) and concurrent medications were also recorded. The ADL, QOL, and PROM questionnaires used in this study were developed by Tikun Olam Ltd. and have been clinically validated [16,17]. Symptom severity prior to cannabis initiation was rated from 0 (no symptoms) to 3 (severe symptoms), and again at six months. Patients also rated the perceived change in symptoms on a scale of −3 (worsening) to +3 (great improvement) and classified their experience as either positive (yes) or not positive (no).

Detailed information regarding chemotherapy type and timing, concurrent medications, and comorbid neuropathies was not available in this dataset. This limitation is explicitly addressed in the Discussion.

### 2.4. Treatment Protocol

Patients consumed cannabis either as smoked/vaporized flowers or orally as concentrated oil extracts. Various strains were used, including CBD-dominant (e.g., Avidekel), THC-dominant (e.g., Alaska, Erez), and balanced THC/CBD (e.g., Midnight). THC doses ranged from 144 to 900 mg/day, and CBD doses ranged from 0 to 430 mg/day.

### 2.5. Statistical Analysis

Descriptive statistics were used for continuous variables presented as means and standard deviations and for categorical variables presented as frequencies and percentages of the total. An unsupervised machine-learning algorithm based upon the k-means clustering method developed by Hartigan and Wong [18] was used to divide patients into groups based upon their CBD and THC consumption. This method minimizes the total squared distance for each patient to the center of their cluster. Using the average silhouette method of Rousseeuw [19], the most appropriate number of clusters was determined by selecting the k with the highest average silhouette for the best cluster arrangement. THC and CBD clusters were characterized by demographics and baseline clinical parameters using a univariate analysis. Linear, logistic, and Poisson regression analyses were used to evaluate the effects of THC and CBD on symptom severity, the ADL and QOL scores, and the symptom numbers. The models accounted for complex interactions between baseline symptom occurrence and severity, baseline ADL and QOL scores, as well as lower-level interactions and main effects. An interaction term for THC×CBD doses was included to assess synergistic or additive effects; the interaction was determined to be additive based on the logistic regression model. Assumption checks were performed before modeling: residuals were tested for normality using the Shapiro–Wilk test and QQ-plots, heteroscedasticity was tested using the Breusch–Pagan test, and multicollinearity was assessed by variance inflation factors (all VIF <2), confirming model validity.

All statistical analyses, including the k-means function, were conducted using R version 4.2.2 [20] a language and environment for statistical computing developed by the R Core Team (2022) and provided by the R Foundation for Statistical Computing, Vienna, Austria (https://www.R-project.org/, accessed on 25 July 2023). RStudio version 2022.12.0+353 [21], an integrated development environment for R, was also utilized to facilitate our data analysis and reporting processes. The alpha level for statistical significance was set at 0.05.

### 2.6. Ethical Considerations

The study was approved as exempt from the local Institutional Review Board (IRB) review due to the use of anonymized data provided by Tikun Olam. Because the dataset was fully anonymized and retrospective, informed consent was waived in accordance with institutional and international guidelines.

## 3. Results

### 3.1. Patient Population

**Figure 3:**
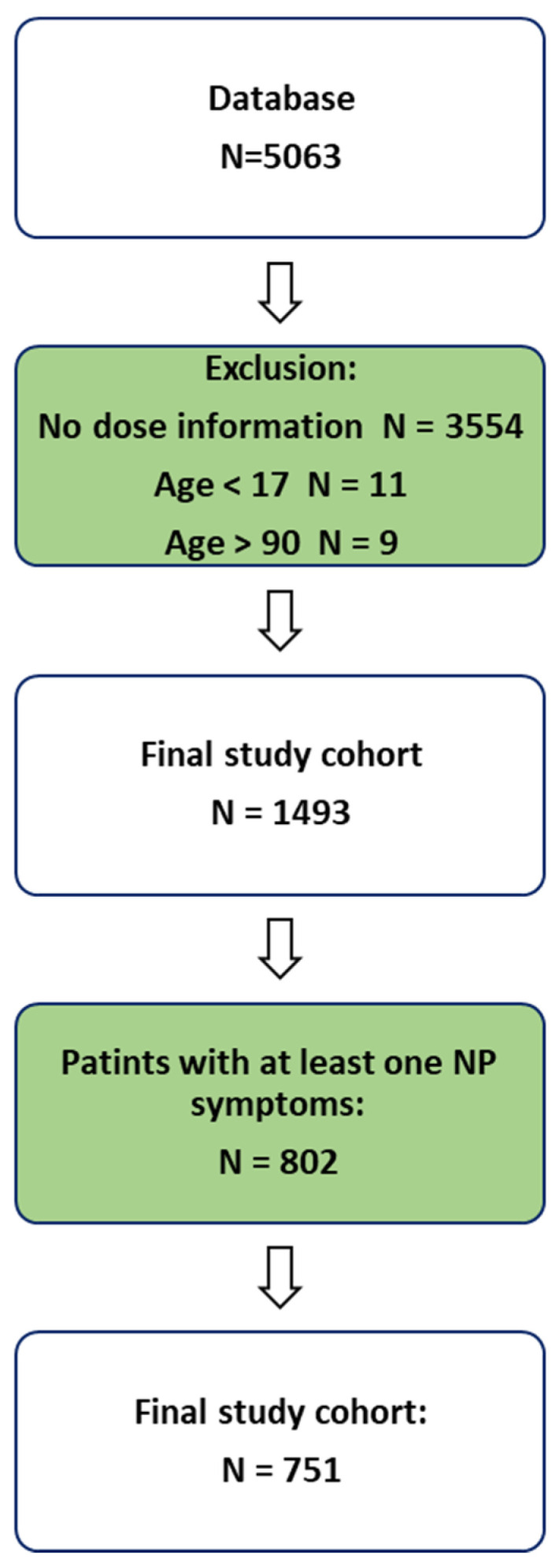
Patient enrollment flowchart.

**Figure 4:**
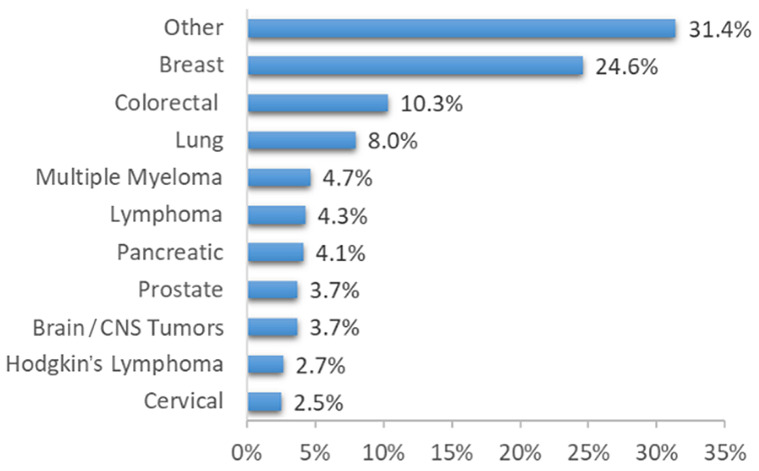
Cancer type distribution within the study cohort.

We identified 802 patients with at least one neuropathic pain (NP) symptom, of whom 751 met all of the inclusion criteria and had full documentation. Figure 3 displays the flowchart illustrating the process of patient enrollment. The cohort consisted of 448 (60%) females. The most common cancer diagnoses were breast cancer (185 patients, 24.6%), colorectal cancer (77 patients, 10.3%), and lung cancer (60 patients, 8%). Table 2 and Figure 4 display the demographic characteristics, medical history, and distribution of cancer types within the cohort.

### 3.2. Cannabis Consumption Characteristics

Patients consumed cannabis either as a smoked or vaporized flower or as concentrated oil, which was taken orally or delivered by vaping. Each patient consumed one or more of several different strains available, including Avidekel, which contains mainly CBD, Alaska and Erez, which contain mostly THC, and Midnight, which contains equal amounts of CBD and THC. The doses of the consumed THC and CBD ranged from 144 to 216 mg/day and from 0 to 36 mg/day, respectively.

### 3.3. Overall Pre- and Post-Treatment of CIPN Symptoms

Prior to treatment, 358 patients (48%) reported a burning sensation, 146 patients (19%) reported a cold sensation, 549 patients (73%) reported paresthesia, and 235 patients (31%) reported numbness. A total of 408 patients (54%) experienced 1 symptom, 194 patients (26%) experienced 2 symptoms, 104 patients (14%) experienced 3 symptoms, and 45 patients (6%) experienced all 4 symptoms. At the end of six months of treatment, improvement in symptom severity was highest for paresthesia, with 320 (43%) patients reporting improvement, followed by 217 (29%) patients reporting improvement in burning sensation, and 112 (15%) patients reporting improvement in numbness. The least symptom improvement was reported for the sensation of cold, with only 67 patients (8.9%) experiencing improvement.

**Figure 5:**
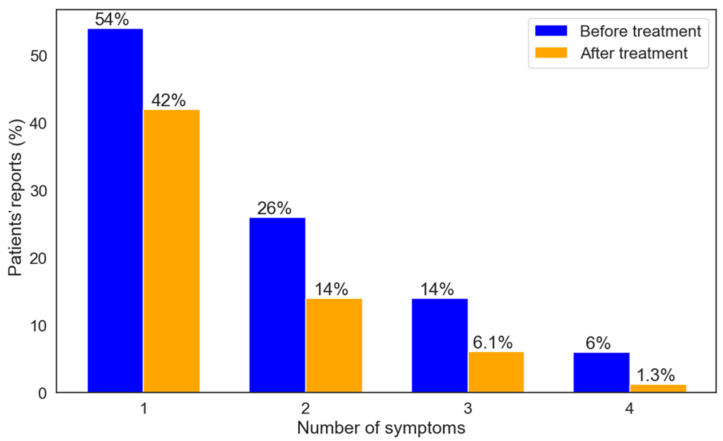
Proportions of patients per number of symptoms pre-treatment and number of symptoms that improved after six months.

A total of 314 (42%) patients reported improvement in 1 symptom, 112 (15%) reported improvement in 2 symptoms, 46 (6.1%) reported improvement in 3 symptoms, and 10 (1.3%) patients reported improvement in 4 symptoms. The reduction in the number of symptoms experienced before and after the treatment is presented in Table S1. Figure 5 shows the percentage of patients who reported the number of symptoms they had at the start of their study entry, as well as the number of symptoms that improved after six months. A total of 572 (78%) patients reported improvement in QOL and 270 (38%) reported improvement in ADL.

### 3.4. Pre- and Post-Treatment Measures of CIPN Symptoms by Cluster Analysis

There were 620 patients in the CBD-high/THC-low cluster and 131 patients in the THC-high/CBD-low cluster. One-half of the patients in the CBD-high/THC-low cluster had CBD levels of up to 430 mg/mL, 50% had no CBD, and THC levels ranged from 0.3 to 236 mg/mL. The THC levels of patients in the THC-high/CBD-low cluster ranged from 240 to 900 mg/mL, and there was no CBD in 85% of the patients and 0-180 mg/mL CBD in the remainder. Prior to treatment, 73% of patients in the THC-high/CBD-low cluster reported paresthesia, 58% reported burning sensation, 39% reported numbness, and 31% reported cold sensation. A similar percentage of patients in the CBD-high/THC-low cluster reported paresthesia before treatment (73%), while all other symptoms were reported by significantly lower percentages of patients: specifically, burning sensation by 45% (p = 0.012), cold sensation by 17% (p < 0.001), and numbness by 30% (p = 0.049). In addition, prior to treatment, the CBD-high/THC-low cluster group had significantly fewer symptoms (p < 0.001), significantly lower cumulative severity (p < 0.001), and significantly lower severity for burning and cold sensations (p = 0.009 and p = 0.002, respectively) compared to the THC-high/CBD-low cluster group.

**Figure 6:**
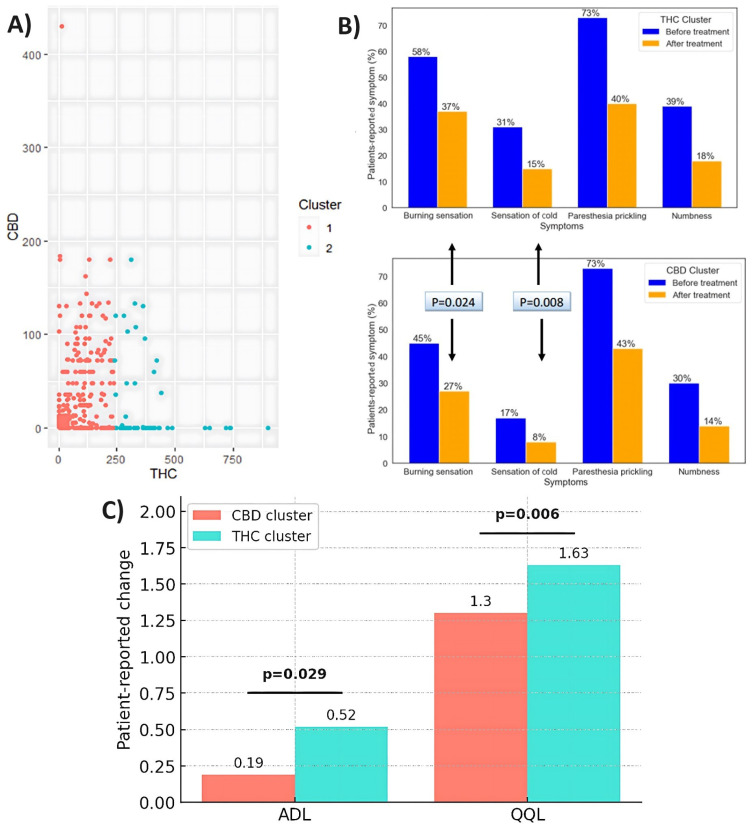
(**A**) Optimal number of clusters based upon THC and CBD doses (mg/day). (**B**) Proportions of patients per pre-treatment symptoms and symptoms that improved after six months. (**C**) Changes in QOL and ADL scores after six months of cannabis treatment.

Patients in both clusters reported improvement in CIPN-related symptoms after six months of treatment (Figure 6A). A significantly greater proportion of patients in the THC-high/CBD-low cluster reported improvement for the burning sensation and cold sensation compared to the CBD-high/THC-low cluster, with a burning sensation at 37% vs. 27%, respectively (p = 0.024) and cold sensation at 15% vs. 8%, respectively (p = 0.008). The proportions of patients reporting paresthesia and numbness after treatment did not differ significantly between clusters, and they were 40% vs. 43% for paresthesia and 18% vs. 14% for numbness in the THC-high/CBD-low cluster compared to the CBD-high/THC-low cluster. Figure 6B shows the percentage of patients who reported the symptoms they had at study entry and the symptoms that improved after six months within each cluster.

### 3.5. Pre- and Post-Treatment Measures of QOL and ADL by Cluster Analysis

Prior to treatment, the CBD-high/THC-low cluster group exhibited significantly higher scores than the THC-high/CBD-low cluster in terms of QOL and ADL (p = 0.011 and p = 0.002, respectively). Following treatment, both QOL and ADL scores showed improvement. The THC-high/CBD-low cluster group demonstrated significantly greater improvements in QOL and ADL compared to the CBD-high/THC-low cluster group (p = 0.006 and p = 0.029, respectively). Figure 6C presents the changes over time in QOL and ADL scores in each cluster.

### 3.6. The Effect of THC and CBD Dosage on Symptom Improvement

**Figure 7:**
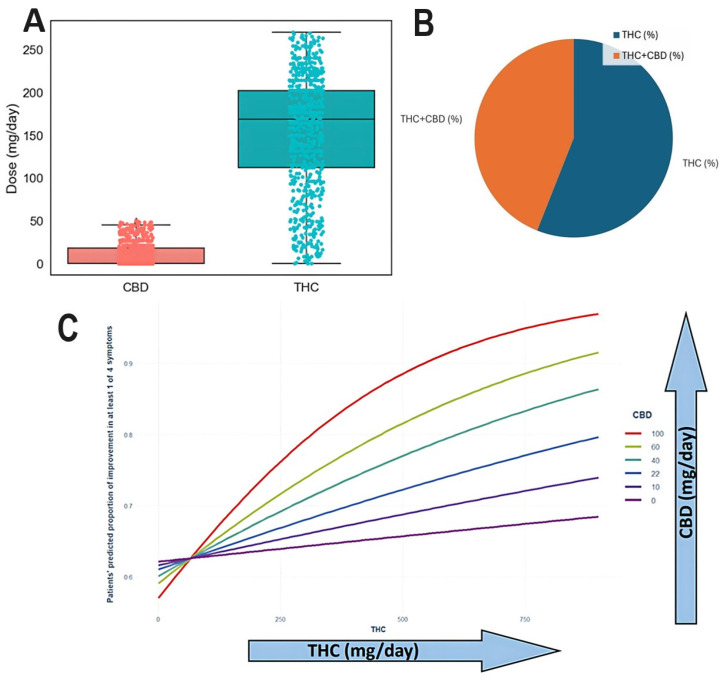
(**A**) Range of CBD and THC doses in the study population. (**B**) Of patients, 56% contained THC alone while 44% contained both THC and CBD. (**C**) Improvement in CIPN-related symptoms by cluster (THC-high vs. CBD-high). Burning sensation and cold sensation improvements are presented with statistical annotations (p = 0.02 and p = 0.008, respectively). The THC × CBD interaction was determined to be additive based on logistic regression modeling.

The range of CBD and THC dosages in the study population was 0-36 mg/day of CBD and 144-216 mg/day of THC (Figure 7A). THC was the sole substance received by 56% of the sample, while 44% received a combination of both (Figure 7B). Patient proportion improvement in at least one symptom was significantly associated with increasing THC and CBD dosages (p < 0.0001). The additive effect of CBD on THC-induced improvement of at least one symptom is illustrated in Figure 7C.

## 4. Discussion

The endocannabinoid system that includes endogenous cannabinoids and cannabinoid receptors [11] regulates pain-related processes [10]. This system is highly integrated within the nervous system circuitry and has an effect upon multiple signaling pathways, as well as being affected by these pathways [11]. Disruption of the endocannabinoid system can contribute to persistent pain [10]. Cannabis strains contain various cannabinoids at different concentrations that bind to cannabinoid receptors found in the brain and peripheral areas [10]. We had previously shown that the use of cannabis can alleviate symptoms of CIPN in patients undergoing chemotherapy [15]. In order to further substantiate the efficacy of medical cannabis and to enhance our understanding of pharmacodynamics, we now retrospectively analyzed variations in THC and CBD doses and their correlation with CIPN symptom alleviation. Our results showed an improvement in all symptoms after six months of uninterrupted cannabis consumption, as well as an overall improvement in ADL and QOL in both groups. We also found that higher doses of both THC and CBD were associated with greater improvement in symptoms.

CB1 and CB2 are the most studied cannabinoid receptors in the human body [11]. CB1 is widely expressed in regions of the brain, including those associated with pain processing and modulation [5,7,9], while CB2 is primarily found in immune cells and in the spleen [6,7,9]. THC is a partial agonist for CB1 and CB2 receptors [5,9]. It produces a psychoactive effect, as well as analgesic, anti-inflammatory, and anti-oxidant effects [6]. In contrast, CBD does not directly interact with the endocannabinoid system due to its low affinity for CB1 and CB2 receptors. However, it may act as a non-competitive inverse agonist, blocking the activation of these receptors [11].

Our findings carry several potential clinical implications. The significant improvement in CIPN symptoms, ADL, and QOL, particularly in the THC-high cluster, supports the clinical use of medical cannabis as a complementary therapeutic option for patients with chemotherapy-induced neuropathy who experience limited relief from standard therapies. The dose-dependent effect of both THC and CBD observed in this study suggests that individualized dose titration, based on patient-reported symptom profiles, may optimize therapeutic benefit while minimizing adverse effects. Moreover, the observed improvement in functionality (ADL) underscores the potential of cannabis to improve daily living and overall patient well-being, aspects often overlooked in traditional CIPN management. Finally, given the additive effect of CBD on THC-induced symptom improvement, clinicians may consider strain selection and ratio adjustment as part of a personalized treatment approach.

This study has several limitations that bear mention. First, its retrospective design relies upon pre-existing data, which limits the ability to control for confounding variables and establish definitive causal relationships between cannabis treatment and its outcomes. Additionally, the study was conducted at a single facility in Israel, which may constrain the generalizability of the findings to other regions or healthcare settings. The participants were individuals who were actively seeking cannabis therapy, thereby introducing potential selection bias and reducing the representativeness of the sample relative to the broader patient population. Moreover, the study focused solely upon two primary cannabinoid dosages, and there are numerous active compounds in cannabis-based treatments that could have impacted the outcomes. The unequal sample sizes between the CBD-high (n = 620) and THC-high (n = 131) clusters, as well as overlapping dose ranges, may affect the validity of the observed differences and require cautious interpretation.

Finally, the data that were derived from self-reports by the participants increased the risk of recall bias and potential inaccuracies.

## 5. Conclusions

Cannabis products demonstrated efficacy in alleviating symptoms associated with CIPN and resulted in a reduction in the number of reported symptoms. Improvements in symptoms and in QOL and ADL questionnaire responses were observed when queried after six months of cannabis use. Higher doses of THC showed greater efficacy than lower doses, while gradually increasing doses of both CBD and THC alone and in combination correlated better with symptom improvement.

The observed dose–response relationship of both THC and CBD highlights the need for prospective controlled trials to establish optimal cannabinoid ratios for specific symptom clusters, such as burning or cold sensations. Future studies should also aim to evaluate the long-term safety and efficacy of cannabis in oncology patients, as well as explore mechanistic pathways linking cannabinoid receptor activation to neuroprotection and anti-inflammatory effects in CIPN. Personalized treatment strategies, incorporating cannabinoid pharmacogenetics and symptom-driven dose titration, should be further investigated to better integrate medical cannabis into standard supportive oncology care.

## Figures and Tables

**Table 1:** Bioactive compounds in cannabis relevant to CIPN.

Compound	Molecular Structure	Mechanism of Action	Therapeutic Effects
Tetrahydrocannabinol (THC)	THC Structure	Partial agonist at CB1 and CB2 receptors	Analgesic, anti-inflammatory, antioxidant, psychoactive
Cannabidiol (CBD)	CBD Structure	Low affinity for CB1/CB2; acts as non-competitive inverse agonist; modulates other receptors	Anxiolytic, antipsychotic, antiepileptic, anti-inflammatory

Note: Zhornitsky and Potvin (2012) [11], Abrams (2022) [7], Whitcomb et al. (2020) [6], and Zylla et al. (2021) [12].

**Table 2:** Patient characteristics.

Overall (n = 751)	No.	%
Age, year M (SD) Median Range	51.8 (17.3) 48.2 17.4–89.4	
Sex		
Female	448	60%
Male	303	40%
Indication for cannabis		
Cancer-related pain	372	50%
Chemotherapy-related side effect	338	45%
Compassionate use	3	0.40%
Depression	1	0.10%
Pain	37	4.90%
Cigarette smoking M (SD) Median Range	0.3 (0.4) 0.0 0.0–0.1	

## Data Availability

The data presented in this study are available on request from the corresponding author. The data are not publicly available due to privacy and ethical restrictions.

## Supplementary Materials

The following supporting information can be downloaded at: https://www.mdpi.com/article/10.3390/biomedicines13081921/s1, Table S1. Reported severity grades of CIPN-related symptoms before and after six months of cannabis treatment.
