# Supplementary material for: Comparative Effects of THC and CBD on Chemotherapy-Induced Peripheral Neuropathy: Insights from a Large Real-World Self-Reported Dataset
Source: Biomedicines. 2025 Aug 6;13(8):1921. doi: 10.3390/biomedicines13081921 (PMC12383924; doi:10.3390/biomedicines13081921)
Supplement: Supplementary file 1 [file biomedicines-13-01921-s001.zip › biomedicines-3764836-supplementary.pdf]

Table S1. Reported severity grades of CIPN-related symptoms before and after six months of cannabis treatment.

Symptom	Mean	SD	Median	Min	Max
Before					
Burning sensation	1.05	1.22	0	0	3
Sensation of cold	0.33	0.73	0	0	3
Paresthesia (prickling)	1.4	1.08	1	0	3
Numbness	0.55	0.91	0	0	3
After 6 months					
Burning sensation change	0.66	1.11	0	−1	3
Sensation of cold change	0.21	0.72	0	−2	3
Paresthesia (prickling) change	0.88	1.22	0	−3	3
Numbness change	0.31	0.85	0	−3	3
